# Yellow Fever in Galveston

**Published:** 1885-12

**Authors:** 


The Yellow Fever in Galveston.—The English steamship Wivanhoe, from Liverpool, via Jamaica, arrived at Galveston on 11th November.

Two days out from Jamaica, a case of fever developed which, in four days, terminated fatally, the man throwing up black vomit. These facts having been communicated to the Quarantine Officer at Galveston, the steamship was held in quarantine to await orders from the State Health Officer at Austin. This officer gave instructions to hold the ship five days to see if any other cases occurred. On Nov. 16, the steamer was allowed to proceed up to her wharf at the city. Two days later, Dr. A. F. Sampson was called to see one of the crew, and suspecting yellow fever, he summoned the Quarantine Officer and the City Physician, Drs. Blount & Cook, who confirmed the diagnosis; whereupon the vessel was immediately remanded into quarantine; and upon telegram from Dr. Blount, State Health Officer R. M. Swearingen visited the patient and concurred in the diagnosis. The patient died two days later. Up to the present writing, Dec. 10th, no other case has been reported, and the instructions given by the Health Officer are to release the vessel at the expiration of ten days from the date of the last death on board.

